# Reproduction, Embryonic Development, and Maternal Transfer of Contaminants in the Amphibian *Gastrophryne carolinensis*

**DOI:** 10.1289/ehp.8457

**Published:** 2005-12-08

**Authors:** William Alexander Hopkins, Sarah Elizabeth DuRant, Brandon Patrick Staub, Christopher Lee Rowe, Brian Phillip Jackson

**Affiliations:** 1 Savannah River Ecology Laboratory, University of Georgia, Aiken, South Carolina, USA; 2 Department of Fisheries and Wildlife Sciences, Virginia Polytechnic Institute and State University, Blacksburg, Virginia, USA; 3 Chesapeake Biological Laboratory, University of Maryland Center for Environmental Science, Solomons, Maryland, USA; 4 Departments of Chemistry and Earth Sciences, Dartmouth College, Hanover, New Hampshire, USA

**Keywords:** amphibian, coal combustion, development, embryo, maternal transfer, mercury, reproduction, selenium, strontium

## Abstract

Although many amphibian populations around the world are declining at alarming rates, the cause of most declines remains unknown. Environmental contamination is one of several factors implicated in declines and may have particularly important effects on sensitive developmental stages. Despite the severe effects of maternal transfer of contaminants on early development in other vertebrate lineages, no studies have examined the effects of maternal transfer of contaminants on reproduction or development in amphibians. We examined maternal transfer of contaminants in eastern narrow-mouth toads (*Gastrophryne carolinensis*) collected from a reference site and near a coal-burning power plant. Adult toads inhabiting the industrial area transferred significant quantities of selenium and strontium to their eggs, but Se concentrations were most notable (up to 100 μg/g dry mass). Compared with the reference site, hatching success was reduced by 11% in clutches from the contaminated site. In surviving larvae, the frequency of developmental abnormalities and abnormal swimming was 55–58% higher in the contaminated site relative to the reference site. Craniofacial abnormalities were nearly an order of magnitude more prevalent in hatchlings from the contaminated site. When all developmental criteria were considered collectively, offspring from the contaminated site experienced 19% lower viability. Although there was no statistical relationship between the concentration of Se or Sr transferred to eggs and any measure of offspring viability, our study demonstrates that maternal transfer may be an important route of contaminant exposure in amphibians that has been overlooked.

Amphibian populations around the world are declining at alarming rates, but the causes of most declines are unknown. Habitat destruction and alteration are most likely the largest contributors to population declines, but other factors such as introduction of exotic species, emerging diseases, global climate change, and environmental contamination likely influence population viability (Collins and Storfer 2003; Stuart et al. 2004). The importance of environmental contamination in population declines is poorly understood, partly because of the dearth of information on effects of contaminants on responses of individuals (e.g., reproduction) that directly influence the viability of natural populations.

Exposure of sensitive embryonic life stages to contaminants may be an important mechanism of impaired reproductive success in amphibians. Developmental pathways in embryonic amphibians can be altered when embryos are exposed to contaminants via two primary mechanisms: uptake from their surroundings (water) and transfer from females to offspring. Most of what is currently known about the effects of contaminants on amphibian development is derived from studies in which embryos are exposed via water. In fact, aqueous exposure has formed the foundation for the only widely accepted standardized amphibian toxicity test (frog embryo teratogenesis assay-*Xenopus*) [American Society for Testing and Materials (ASTM) 1998; Bantle et al. 1991]. In contrast, maternal transfer has been an understudied mechanism of reproductive impairment in amphibians. One previous study demonstrated that amphibians partition significant quantities of cadmium in reproductive tissues (in follicles and eggs before oviposition), suggesting the potential for maternal transfer to offspring (Linder et al. 1998). Kadokami et al. (2004) went a step further when they demonstrated that frogs can maternally transfer organic contaminants to their eggs. However, no studies have examined the effect of maternal transfer on reproduction and development in amphibians, despite well-known effects of maternally derived contaminants in all other major vertebrate lineages (e.g., Skorupa 1998). Ironically, for many of the most toxic contaminants (e.g., lipophilic compounds and mercury), maternal transfer may represent the most important route of exposure for amphibian embryos because water concentrations of these substances are generally low.

The purpose of the present study was to determine whether amphibians maternally transfer metals and metalloids to their eggs and whether such transfer has implications for reproductive success by affecting offspring viability. We focused on anurans inhabiting a site contaminated by a coal-burning power plant because these sites are a major global source of selenium contamination (Lemly 1996; Rowe et al. 2002). Selenium is a contaminant that is readily transferable from mother to egg, highly teratogenic, and associated with population declines in other vertebrates (Lemly 1996; Ohlendorf 2003). Previous work in the contaminated study site suggests that it is inhabited by at least 22 species of amphibians, but recruitment of juveniles from the contaminated wetlands appears low (Rowe and Hopkins 2003). We hypothesized that disruption of normal development due to maternal transfer of Se and/or other elements may partially underlie these observations.

## Materials and Methods

### Study species and sites.

The species chosen for study, the eastern narrow-mouth toad (*Gastrophryne carolinensis*), is a common constituent of amphibian communities in the southeastern United States. Like many other anurans with complex lifecycles, *G. carolinensis* adults are terrestrial and congregate near water during the breeding season (May–September in South Carolina). The species is particularly successful at breeding in ephemeral water bodies. Adult *G. carolinensis* are small, highly fossorial, and feed predominately on ants, termites, and beetles. Unlike other anurans in the southeastern United States, the larvae of *G. carolinensis* filter-feed plankton from the water column (Pechmann 1994).

Toads were collected from two sites on the Savannah River Site, near Aiken, South Carolina. The contaminated site was the D-area electric power generation facility, which has associated with it at least 126 ha of land affected by historical and current disposal of sluiced coal combustion wastes (CCWs). Current disposal of CCWs occurs in large settling basins, one of which has partially filled and become extensively revegetated. The revegetated portion of this CCW basin is centrally located within power plant disposal operations and was the focal sampling area for this study. The reference site was Ginger’s Bay, a 1.5-ha Carolina bay (i.e., isolated freshwater wetland) located approximately 13.5 km from the polluted site. The bay has been extensively sampled for amphibians in the past and has no deliberate anthropogenic input of contaminants (Hopkins et al. 2000).

### Collection, breeding, and assessment of development.

Animals used in this study were treated humanely and with regard for the alleviation of suffering according to approved protocols of the University of Georgia’s Animal Care and Use Committee. Adult male and female *G. carolinensis* were collected during the breeding season (22 May–7 July 2003) at both sites using drift fences. Upon collection, adults were transported to an array of outdoor mesocosms (*n* = 76) up to 3,000 L in volume. One female and one or two males collected from the same site were placed within each mesocosm and allowed to breed. Each mesocosm was slightly slanted such that one end of the mesocosm contained at least 10 cm well water, but the remaining portion of the mesocosm’s interior base was dry and covered with leaf litter and debris collected from the reference site. Mesocosms were checked every morning for breeding activity, and fresh egg masses were removed immediately upon discovery.

Egg masses (*n* = 20 and 37, contaminated site and reference site, respectively) were returned to the laboratory, where they were enumerated and a subsample of 100 fertile eggs were set aside for assessments of development. These 57 subsamples were then allowed to develop in 3.8 L dechlorinated tap water (dissolved oxygen = 9.03 ± 0.10 mg/L; pH, 8.17 ± 0.02; conductivity = 142 ± 1.96 μS/cm) for 96 hr at 25°C. After 96 hr, hatchlings from each subsample were counted to quantify hatching success. Hatchlings were then placed in a 60 × 15 mm glass Petri dish filled with dechlorinated tap water.

Each hatchling was encouraged to swim by gently prodding with a plastic pipette. Swimming behavior was classified as abnormal if individuals failed to respond after repeated prods, failed to maintain upright posture, or swam erratically in circles. Normal and abnormal swimmers were separated and individuals were placed in 20-mL vials containing MEMFA (0.1 M MOPS, pH 7.4, 2.0 mM EGTA, 1.0 mM MgSO_4_, 3.7% formaldehyde). After 2 hr of fixation under continual rotation, the MEMFA solution was completely exchanged with 100% ethanol, and samples were stored at 4°C for subsequent morphologic assessments. Each hatchling was examined under 6×–25× magnification for morphologic abnormalities according to the methods of Bantle et al. (1991) and ASTM (1998). Morphologic abnormalities were classified as edema or swelling, skin blistering, craniofacial malformations, and/or four types of axial malformations (dorsal flexure, lateral flexure, wavy tail, and axial shortening).

### Trace element analysis.

Immediately after removing the 100 egg subsets from the clutches for developmental assessments, we froze the remaining eggs from 10 and 18 of the clutches (contaminated and reference site, respectively) at −60°C for subsequent chemical analysis. We held the females that produced these clutches for 48 hr after oviposition to ensure that they were postabsorptive and then anesthetized them before freezing (−60°C). Adult toads and their eggs were later prepared for contaminant analysis according to the methods of Snodgrass et al. (2004, 2005).

We digested approximately 20–180 mg lyophilized adult toad carcasses and eggs by adding trace metal grade nitric acid (70% HNO_3_, 2.5–5.0 mL) to samples before digestion in a microwave (MDS 2000; CEM Corp., Matthews, NC) with heating steps of 60, 60, 70, and 80% microwave power for 10, 10, 15, and 20 min, respectively. After HNO_3_ digestion, we added 1.0 mL hydrogen peroxide to the samples and microwaved them at the same power and duration as the HNO_3_ digestion. After digestion, we brought samples to a final volume of 10 mL with 18 MΩ deionized water. Soil and water samples were also collected (*n* = 3/matrix/breeding site). Soil samples (~ 200 mg) were oven dried before digesting with 9.0 mL HNO_3_ and 1.0 mL H_2_O_2_ as described above, and brought to a final volume of 50 mL with deionized water before analysis. Water samples (10 mL) were filtered (0.44 μm) and acidified before analysis.

We performed element analysis (arsenic, Cd, copper, iron, Hg, nickel, Se, strontium, vanadium, and zinc) by inductively coupled plasma mass spectrometry (Perkin Elmer, Norwalk, CT) on samples diluted 1:10 with deionized water. We calibrated the instrument daily using calibration standards covering a range of 1–500 μg/L prepared by serial dilution of NIST traceable primary standards (National Institute of Standards and Technology, Gaithersburg, MD). For quality control purposes, we included certified reference material (TORT-2; National Research Council of Canada, Ottawa, Canada) and reagent blanks in the digestion and analysis procedure. Mean percent recoveries for elements in tissue reference material ranged from 84 to 106%. Detection limits (micrograms per liter) in water samples were as follows: As, 0.41; Cd, 0.09; Cu, 1.59; Fe, 2.40; Hg, 0.11; Ni, 0.25; Se, 0.13; Sr, 0.03; V, 0.06; Zn, 5.13. Detection limits (nanograms per gram) in soil and tissue were as follows: As, 0.13; Cd, 0.17; Cu, 0.55; Fe, 19.67; Hg, 0.11; Ni, 2.21; Se, 0.26; Sr, 0.14; V, 0.12; Zn, 5.15. Concentrations of elements in tissues and soils are reported on a dry mass basis.

### Statistical analysis.

In all statistical comparisons, significance was assessed at *p* < 0.05, but in cases where multiple comparisons were made, we adjusted *p*-values using a sequential Bonferroni adjustment to maintain an experiment wide error rate of α = 0.05. We used log or angular transformations to better approximate assumptions of parametric statistical models.

Trace element concentrations in water, soil, adult females, and freshly laid eggs were compared between sites using either a series of analyses of variance (ANOVAs) or a multivariate analysis of variance (MANOVA). For soils and water, small sample sizes (*n* = 3/site) prohibited us from using multivariate analyses. Therefore, ANOVAs were used for comparison of 10 elements between sites. Only one soil concentration (Hg in a single reference soil sample) was below the detection limit (BDL) and was replaced with 50% of the analytical detection limit. For tissues from adults, > 50% of Ni and As concentrations were BDL; therefore these elements were not compared between sites. Hg and V concentrations were BDL in two and eight adults, respectively, collected at the reference site; these values were replaced with 50% of the detection limit for statistical comparisons. Adult tissue concentrations were then compared by MANOVA. For egg masses, > 50% of Ni, As, Cd, and Hg concentrations were BDL and were therefore not compared statistically. One egg mass collected from the contaminated site had low V concentrations and was replaced with 50% of the detection limit before inclusion in a multivariate model. We used logistic regression to model how Se concentration in eggs influenced the probability of Hg transfer to eggs at levels above our detection limits. To examine the relationship between contaminant concentrations in female carcasses and their eggs, we used linear regression of log-transformed data both within and between sites. We also used Pearson correlation coefficients to describe relationships between elements that were maternally transferred.

For elements that were maternally transferred, total preovipositional body burdens (micrograms) were reconstructed using individual tissue (egg and whole body) element concentrations and tissue dry masses. Total body (egg + carcass) burdens (dry) were compared between sites using ANOVA. The percentages of total body burdens deposited in eggs were then calculated and compared using ANOVA.

The mass and snout–vent length (SVL) of females was compared between sites using MANOVA, and Pillai’s trace statistic was used to assess statistical significance. Clutch size of females was compared between sites using ANOVA. Body size was not included as a covariate in this analysis because there was no relationship between female size and clutch size.

The percentages of individuals from the 57 clutch subsets that hatched, exhibited abnormal morphology, and swam abnormally were compared between sites using a series of Wilcoxon two-sample tests. In an effort to estimate the overall viability of each clutch, we then combined the number of individuals not hatching, the number of individuals exhibiting morphologic abnormalities, and the number of individuals exhibiting abnormal swimming behavior (but were otherwise morphologically normal) to calculate a viability index. The percentage of viable hatchlings produced from the clutch subsets were compared between sites using a Wilcoxon two-sample test.

To determine whether there was a relationship between maternal transfer of contaminants and developmental parameters (hatching, swimming, malformations, and viability), we conducted a series of linear regressions. Iterations of these models were conducted using data only for the contaminated site as well as for both study sites pooled.

## Results

### Contaminant concentrations.

As we expected, soils from the contaminated site had significantly higher concentrations of several trace elements compared with the reference site (Table 1). Cu, Ni, Se, Sr, and V concentrations were three to eight times higher in contaminated soils than in reference soils (in all cases *p* ≤ 0.001). Arsenic concentrations were more than 60 times higher in contaminated soils compared with reference soils (*p* < 0.001). In contrast, soil concentrations of Hg, Zn, and Fe were similar between sites (*p* ≥ 0.08). Although Cd concentrations were twice as high in contaminated soils than in reference soils (*p* = 0.027), the difference was not statistically significant after Bonferroni adjustment.

Water concentrations of contaminants also varied greatly between sites (Table 1). Cu, Fe, and Zn concentrations were significantly higher in the reference site (in all cases *p* ≤ 0.004). In contrast, dissolved concentrations of As, Ni, Se, Sr, and V were higher in the contaminated site compared with the reference site (in all cases *p* ≤ 0.005). Cd concentrations were similar in the water at each site (*p* = 0.016), and Hg concentrations were BDL in all water samples.

Adult females collected at the contaminated site had significantly higher whole-body concentrations of trace elements compared with females from the reference site (MANOVA, *F*_8,19_ = 12.33; *p* < 0.001). Most notably, accumulation of V, Sr, and Se was 3, 7, and 22 times higher in females from the contaminated site compared with the reference site (Table 2). However, the opposite was true for Hg. Females collected from the reference site had whole-body Hg concentrations that were 60% higher than in females from the contaminated site.

**Figure 1:**
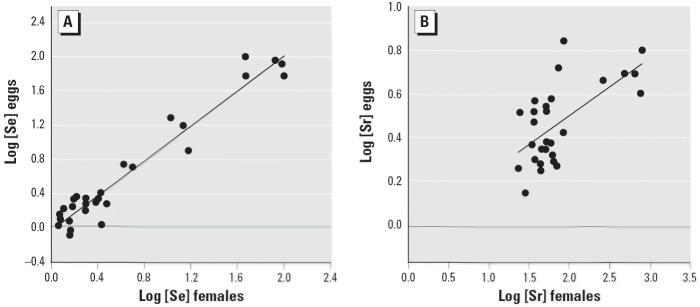
Relationship between Se (*A*) and Sr (*B*) concentrations (μg/g dry mass) in female *G. carolinensis* and their eggs. Females were collected from both contaminated and reference sites but bred under controlled, uncontaminated conditions. Female concentrations were determined 48 hr after oviposition. (*A*) *y* = 1.0255*x* − 0.0448, *r*^2^ = 0.9415. (*B*) *y* = 0.2634*x* − 0.0237, *r*^2^ = 0.4026.

Of the three elements (Se, Sr, and V) accumulated by females at the contaminated site, Sr and Se were also transferred to their eggs at greater concentrations than to reference eggs (*p* ≤ 0.01; Table 2). Concentrations of Se and Sr in eggs were positively correlated (*r* = 0.682). Se concentrations in eggs ranged from 5.37 to 99.18 μg/g dry mass and were strongly influenced by the concentration of Se in female tissues (*r*^2^ = 0.94, *p* < 0.001; Figure 1A). The relationship between Sr concentration in females and their eggs was also strong (*r*^2^ = 0.40, *p* < 0.001; Figure 1B) but was more variable than the relationship observed for Se. In contrast, maternal transfer of V was low and similar between sites (*p* = 0.14).

**Figure 2:**
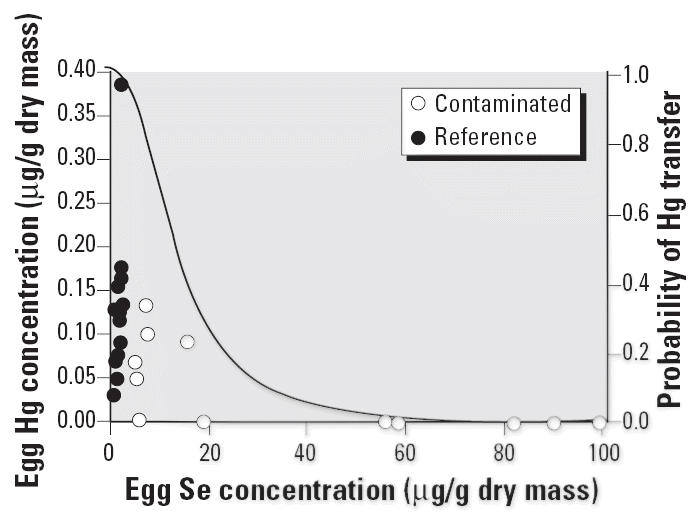
Relationship between Se and Hg concentrations maternally transferred to eggs. The solid line represents the probability (logistic regression model) of transferring Hg to eggs at concentrations above detection limits (0.11 ng/g).

Although Hg concentrations were generally low in all samples, Hg concentrations in eggs from contaminated females spanned orders of magnitude, and mean concentrations were much lower than in eggs from reference females. However, 7 of 10 egg masses from the contaminated site had Hg concentrations BDL, preventing rigorous statistical comparisons between sites. Logistic regression indicated that the Se concentration in eggs strongly influenced (*p* < 0.01) the probability of Hg being transferred to eggs at concentrations above our detection limits (Figure 2).

**Figure 3:**
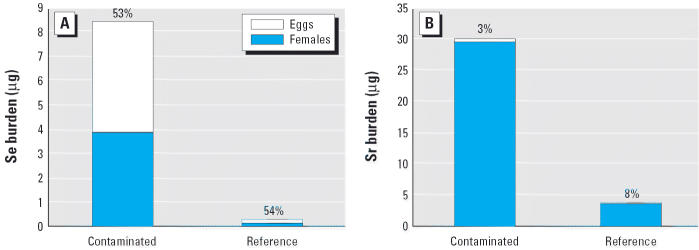
Total body burden (μg) of Se (*A*) and Sr (*B*) in female *G. carolinensis* collected from both contaminated and reference sites. Total body burden is partitioned between eggs and the postovipositional female carcass. Sr in reference eggs is not visible on the graph because these eggs only contained an average of 0.29 μg Sr. Percentages above bars signify the proportion of the total body burden that was transferred to eggs at oviposition.

Reconstruction of preovipositional body burdens and the percentage of this burden lost at oviposition revealed different partitioning patterns for Se and Sr. Preovipositional body burdens of Se were > 25 times higher in females from the contaminated site compared with the reference site (hereafter all data are presented as mean ± 1 SE: 8.45 ± 2.39 vs. 0.33 ± 0.02 μg Se, respectively; *F* = 96.83, *p* < 0.001). However, the proportion of the total body Se burden that was transferred to eggs was high and equivalent between sites (53–54%, *p* = 0.96; Figure 3). In contrast, preovipositional body burdens of Sr were approximately 10 times higher in females from the contaminated site compared with the reference site (30.23 ± 9.03 vs. 3.83 ± 0.26 μg Sr, respectively; *F* = 30.04, *p* < 0.001), but the total amount of Sr maternally transferred was similar between sites (0.40 ± 0.06 vs. 0.29 ± 0.03 μg Sr, respectively; *F* = 3.16, *p* = 0.09). Thus, the proportion of total body Sr burden that was transferred to eggs was low (~ 3–8%) but was more than two times higher in females from the reference site compared with the contaminated site (*F* = 16.95, *p* < 0.001; Figure 3).

### Reproduction and development.

Adult females collected from the reference site (SVL = 29.1 ± 0.26 mm; mass = 2.19 ± 0.07 g) were significantly larger (Pillai’s trace = 0.246; *F*_2,54_ = 8.83; *p* < 0.001) than adult females from the contaminated site (SVL = 27.4 ± 0.39 mm; mass = 1.76 ± 0.07 g). However, clutch size was similar between sites (*F* = 0.16; *p* = 0.69; 674 ± 50 and 598 ± 41 eggs, reference and contaminated site, respectively). No relationship was detected between body size and clutch size for reference site only (*r*^2^ < 0.001, *F* < 0.01, *p* = 0.95), contaminated site only (*r*^2^ = 0.105, *F* = 2.12, *p* = 0.16), or both sites combined (*r*^2^ = 0.012, *F* = 0.65, *p* = 0.42).

Embryonic development was impaired in eggs originating from the contaminated site. Hatching success was significantly reduced in eggs from females collected in the contaminated site compared with the reference site (percent hatching = 83 ± 6.8 vs. 93 ± 2.6, respectively; *p* = 0.003). The frequency of developmental abnormalities in hatchlings was significantly higher in the contaminated site than in the reference site (clutch mean percent abnormal = 16.7 ± 5.7 vs. 10.5 ± 3.6, respectively; *p* = 0.019). Axial malformations were the most commonly observed abnormalities in both sites (Table 3). Craniofacial abnormalities were rare in reference hatchlings but were quite common among abnormal larvae from the contaminated site. Many individuals that exhibited morphologic abnormalities also displayed impaired swimming behavior. Of the 574 malformed hatchlings from the 57 clutches examined, 88% exhibited abnormal swimming behavior. In contrast, only 3% of 4,528 individuals with normal morphology swam abnormally. Frequency of abnormal swimming was higher among hatchlings from the contaminated site compared with the reference site (percent abnormal swimming: 19.1 ± 6.3 vs. 12.4 ± 4.2, respectively; *p* = 0.009). When hatching, morphology, and swimming were considered together as a viability index, the percentage of viable offspring was significantly higher in the reference site (82.2 ± 4.8) compared with the contaminated site (66.6 ± 8.2; *p* = 0.003).

Based on our statistical models, there was no relationship between Se concentration in eggs and hatching success, malformation frequency, percentage of individuals exhibiting abnormal swimming behavior, or total viability, regardless of whether the contaminated site was considered independently or if the sites were pooled (in all cases *r*^2^ < 0.20, *p* > 0.19). Similarly, Sr concentration in eggs was not statistically related to any of the measured developmental parameters (in all cases *r*^2^ < 0.08, *p* > 0.14).

## Discussion

Our study definitively demonstrated that amphibians, like other vertebrates, are capable of transferring potentially harmful concentrations of inorganic contaminants to their eggs. Most notably, we described maternal transfer of high concentrations of Se that were strongly related to the concentration of Se accumulated in female tissues. In addition, we documented a significant reduction in offspring viability in frogs collected near a coal-burning power plant compared with a reference site. Our findings highlight the importance of further research on maternal transfer of contaminants in amphibians, especially as it relates to viability of natural populations.

### Maternal transfer.

Although females inhabiting the contaminated site accumulated several trace elements in their tissues, only Se and Sr were maternally transferred to eggs in appreciable quantities. Unlike some lipophilic compounds, Se and Sr were likely transferred from mother to offspring due to their substitution for sulfur and calcium (respectively) in egg components (e.g., Kroll and Doroshov 1991; Wysolmerski 2002). Because Se is also an essential micronutrient, small additional quantities of this element may have been transferred to eggs as an active constituent of important seleno-proteins. Maternal transfer of Sr to eggs was much more conservative than Se transfer, resulting in egg Sr concentrations 20–80 times lower than Sr concentrations in the female carcass. In contrast, maternal transfer of Se resulted in egg concentrations similar to concentrations in the female carcass. Functional relationships between Se concentrations in female carcass and eggs have not been well described in the literature for fish or other wildlife. However, comparison of Se concentrations in whole postpartum female mosquitofish and their fry indicated that offspring had higher concentrations of Se than their mothers (Saiki et al. 2004). More directly analogous comparisons have been made for Hg; strong linear relationships have been observed between Hg concentrations in female fish and their eggs (Hammerschmidt and Sandheinrich 2005; Hammerschmidt et al. 1999).

Partitioning of Se and Sr burden (micrograms) between somatic tissues and eggs was different for each element. Regardless of Se exposure history, female frogs partitioned approximately 53% of their total Se burden into their eggs at oviposition. In contrast, the percentage of Sr body burden transferred to eggs was much lower and dependent upon exposure history. Frogs from the reference site proportionally transferred more than twice as much of their Sr burden to eggs compared with frogs from the contaminated site (8% vs. 3%, respectively). The observation that Sr transfer only ranged from 0.29 to 0.40 μg despite a 10-fold range in total body burdens between sites suggests that the upper limit of Sr transfer to eggs may have been approached. In contrast, there was no evidence that maternal transfer of Se was asymptotic. Such partitioning relationships have not been well described for Se or Sr in wildlife. However, a recent study provided a detailed description of Se partitioning among organs in male and female fence lizards (*Sceloporus occidentalis*) fed low (1 μg/g) and high (15 μg/g) Se concentrations (Hopkins et al. 2005). Regardless of the amount of Se fed to lizards, females partitioned approximately 33% of their total Se burden to ovaries, most of which had produced enlarged, yolked follicles at the time they were examined. Because lizards were not permitted to oviposit, it is not known what the total partitioning of Se would have ultimately been to eggs. However, the facts that both *G. carolinensis* and *S. occidentalis* transferred a constant proportion of their total Se burden to eggs or follicles (~ 53% and 33%, respectively) and that the relationship between Se concentrations in female *G. carolinensis* and their eggs was nearly 1:1 suggest that the amount of Se ultimately transferred to young is largely governed by the mass of reproductive tissue produced. Indeed, the dry mass of frog eggs (present study) and lizard follicles (Hopkins et al. 2005) represented 51 ± 0.03% and 25 ± 0.02% of total body dry mass. This observation suggests that the reproductive strategy employed by organisms (e.g., large vs. small clutches) may have important implications for the quantity of Se transferred during each reproductive event.

Se concentrations in female *G. carolinensis* greatly exceeded concentrations measured in other amphibian species previously studied at the same contaminated site, but Sr concentrations were similar among adults of all species examined heretofore. For example, adult southern toads (*Bufo terrestris*) sampled near the power plant had mean Se and Sr concentrations of 17 and 387 μg/g, respectively (Hopkins et al. 1998). More recently, adult *B. terrestris* and leopard frogs (*Rana sphenocephala*) sampled from a contaminated downstream area had mean Se and Sr concentrations up to 7 and 325 μg/g, respectively (Roe et al. 2005). It is unclear why *G. carolinensis* accumulates such high concentrations of Se (42 μg/g) while simultaneously accumulating comparable concentrations of Sr (324 μg/g) compared with other species studied. The most parsimonious explanation may involve the unusual ecology of *G. carolinensis*, which is highly fossorial and feeds primarily on ants, termites, and beetles. It is possible that these habits result in greater trophic exposure to Se than that experienced by adult *B. terrestris* or *R. sphenocephala*. Regardless of the reason for the observed differences in Se accumulation among various species, high interspecies variability highlights the importance of sampling multiple species of amphibians with diverse ecologies when trying to assess risk in contaminated habitats.

Maternal transfer of Se by *G. carolinensis* also greatly exceeded the Se concentrations maternally transferred by female reptiles, birds, and fish studied at the same site. American alligators (*Alligator mississippiensis*) and common grackles (*Quiscalus quiscala*) both transferred Se concentrations of 6–7 μg/g to their eggs, but neither of these studies measured female Se concentrations or rigorously addressed the effects of transfer on reproduction and development (Bryan et al. 2003; Roe et al. 2004). Adult female mosquitofish (*Gambusia holbrooki*) had Se concentrations of 12 μg/g in their carcasses after giving birth to offspring with Se concentrations of 16 μg/g (Staub et al. 2004). Reproductive success and offspring viability in mosquitofish were not affected by maternal transfer of Se, an observation consistent with the known tolerance of mosquitofish to Se (Cherry et al. 1976; Lemly 1985; Saiki et al. 2004).

Finally, patterns of accumulation and maternal transfer of Hg were noteworthy. In contrast to other elements, concentrations of Hg were higher in females and eggs from the reference site compared with the contaminated site. Logistic regression suggested that females that transferred concentrations of Se ≥ 20 μg/g to their eggs had very little (i.e., concentrations BDL) Hg in their eggs. We hypothesize that high concentrations of Se in females from the contaminated site may have inhibited Hg accumulation in female tissues, resulting in low concentrations of Hg available for subsequent transfer to offspring. Although many studies have demonstrated antagonistic bioaccumulation patterns between Se and Hg (Cuvin-Aralar and Furness 1991; Southworth et al. 2000), we know of few studies that have highlighted the important implications of Se–Hg antagonism for maternal transfer of Hg (Heinz and Hoffman 1998).

### Reproductive and developmental effects.

In ecologic terms, maternal transfer of contaminants is important because of effects on reproductive success and ultimately, population viability. We found that breeding adults at the contaminated site experienced a 19% reduction in the percentage of their offspring that were viable compared with adults from the reference site. The abnormalities that contributed to this reduction were consistent with those in fish exposed to seleniferous discharge from other power plants (Lemly 1996).

A perplexing finding from this study was the lack of a relationship between concentrations of Se or Sr transferred to eggs and indices of offspring viability. It is not known whether the concentrations of Sr observed in eggs should be expected to cause abnormalities because little is known about the effects of Sr on early embryonic development in wildlife, although injection studies suggest that high concentrations of Sr can be embryotoxic (Ridgway and Karnofsky 1952). In contrast, Se in the contaminated site was transferred at levels known to be embryotoxic to fish and birds (4–16 μg/g dry mass) (Lemly 1996; Ohlendorf 2003). At the highest Se concentrations observed in frog eggs (i.e., 80–100 μg/g dry mass) offspring viability was quite variable but as high as 95%. There are several possible explanations for not detecting a relationship between Se transfer and offspring viability. From a methodologic perspective, we measured contaminant concentrations in 28 clutches of eggs, but only five of these clutches contained Se concentrations > 20 μg/g dry mass. Thus, our statistical power for detecting functional relationships between concentrations and effects was probably limited within the range of concentrations where effects should be most predominant. From a biologic perspective, it is possible that *G. carolinensis* is more tolerant of Se than most fish and birds studied to date. Such tolerance would not be unprecedented; mosquitofish and cutthroat trout appear extraordinarily tolerant of Se embryotoxicity, remaining asymptomatic even after transferring Se concentrations of approximately 20–80 μg/g dry mass to their young (Kennedy et al. 2000; Saiki et al. 2004). Alternatively, Se partitioning among egg components could have obfuscated our results. Frogs produce a gelatinous jelly coat around their eggs that contains a variety of sulfur-containing mucoproteins and mucopolysaccharides that provide important structural barriers around the embryo essential for fertilization and hatching but are not believed to be nutritive (Duellman and Trueb 1986). Our contaminant analysis of eggs included this jelly coat and may therefore include some Se-containing compounds that were not bio-available to the developing embryo. Last, we cannot rule out the possibility that other contaminants may have been present at the industrial site and contributed to our findings. Although metals and metalloids are believed to be the key toxic constituents of CCW (Rowe et al. 2002), organic contaminants such as polycyclic aromatic hydrocarbons could occur in this complex waste stream. However, available information suggests that the concentrations of these organic materials in CCWs are extremely low [U.S. Environmental Protection Agency (EPA) 1999].

Finally, our findings shed further insight into the ecologic and human health hazards posed by disposal of solid wastes from coal-fired power plants. Many previous studies have demonstrated that amphibian larvae developing in sites contaminated by CCWs bioaccumulate high concentrations of contaminants and exhibit developmental, physiologic, and behavioral abnormalities (reviewed by Rowe et al. 2002). Laboratory studies and transplantation of larvae from reference sites to CCW-contaminated sites confirm these accumulation patterns and reveal additional effects on growth, survival, and key life history characteristics (Rowe et al. 2002; Snodgrass et al. 2004, 2005). Taken together, these experiments also suggest that the severity of these effects may be exacerbated by confounding ecologic factors (e.g., overwintering, per capita resource availability) in the field. The observations that adult female anurans inhabiting these sites can accumulate high concentrations of Se and Sr in their tissues, transfer Se and Sr concentrations to their offspring, and exhibit reductions in offspring viability raises many new questions regarding the impact of aquatic CCW-disposal practices on amphibians. For example, because our study only tracked development for the first 96 hr, the possibility remains that latent effects of maternal transfer may become evident later in development (e.g., Heinz et al. 1989). Further studies designed to track amphibian development through metamorphosis are clearly needed to assess the consequences of maternal transfer to reproductive success and ultimately as one of many important contributing factors to amphibian declines.

## Figures and Tables

**Table 1:** Elemental composition of water and soil collected from the contaminated and reference sites, and laboratory water used for rearing embryos for 96 hr.

		Site water (μg/L)		Soil (μg/g dry mass)	
Element	Lab water (μg/L)	Reference	Contaminated	Reference	Contaminated
As	BDL	1.10 ± 0.03	117.70 ± 5.93	1.74 ± 0.23	114.96 ± 7.90
Cd	0.60 ± 0.01	0.55 ± 0.01	0.71 ± 0.04	0.58 ± 0.11	1.17 ± 0.07
Cu	2.81 ± 0.49	9.39 ± 0.76	4.61 ± 0.39	30.66 ± 4.08	100.17 ± 5.28
Fe	18.10 ± 1.14	736 ± 20.87	111 ± 5.84	11,399 ± 5,326	25,224 ± 3,084
Hg	BDL	BDL	BDL	0.11 ± 0.03	0.20 ± 0.01
Ni	0.49 ± 0.03	1.53 ± 0.16	2.70 ± 0.09	8.94 ± 0.60	46.88 ± 1.38
Se	0.28 ± 0.04	0.19 ± 0.03	3.93 ± 0.14	1.64 ± 0.10	8.25 ± 0.64
Sr	3.38 ± 0.14	11.77 ± 0.36	407.15 ± 22.39	28.88 ± 10.12	222.50 ± 11.20
V	BDL	0.26 ± 0.01	8.34 ± 0.36	13.99 ± 1.25	70.15 ± 1.76
Zn	24.83 ± 4.62	182.05 ± 12.14	41.47 ± 5.25	1047.63 ± 139.58	919.08 ± 13.25

Data are presented as mean ± 1SE; *n* = 3 samples/matrix/site.

**Table 2:** Elemental composition of postovipositional female *G. carolinensis* and their eggs from reference and contaminated sites in South Carolina.

	Females (μg/g dry mass)		Eggs (μg/g dry mass)	
Element	Reference	Contaminated	Reference	Contaminated
As	BDL	BDL	0.74 ± 0.56	BDL
Cd	0.34 ± 0.03	0.44 ± 0.21	BDL	BDL
Cu	4.09 ± 0.17	4.61 ± 0.99	9.30 ± 2.06	7.01 ± 3.47
Fe	442.93 ± 37.50	448.72 ± 109.40	278.29 ± 34.91	195.29 ± 171.35
Hg	0.31 ± 0.04	0.19 ± 0.13	0.11 ± 0.02	BDL
Ni	BDL	BDL	BDL	BDL
Se	1.85 ± 0.14	42.40 ± 38.78	1.63 ± 0.12	43.96 ± 37.62
Sr	44.22 ± 3.24	324.23 ± 303.43	2.68 ± 0.23	4.14 ± 1.73
V	0.09 ± 0.01	0.29 ± 0.22	0.49 ± 0.05	0.43 ± 0.37
Zn	173.97 ± 12.17	181.05 ± 59.72	223.49 ± 41.78	222.71 ± 138.93

Data are presented as mean ± 1SE; *n* = 10 and 18 contaminated and reference, respectively.

**Table 3:** Comparison of specific morphologic abnormalities among abnormal *G. carolinensis* hatchlings collected from females from contaminated and reference sites.

	Percent abnormal individuals^a^			
Site	Edema/swelling	Blisters	Craniofacial	Axial
Reference (*n* = 37 clutches)	17.5	1.8	4.6	84.0
Contaminated (*n* = 20 clutches)	22.8	0	38.8	61.6

a Cumulative percentage of abnormalities exceeds 100% because many individuals displayed multiple morphologic abnormalities.
